# Vaccine effectiveness in preventing deaths in people with severe acute respiratory syndrome due to COVID-19 in Blumenau, Brazil, 2021

**DOI:** 10.1590/S2237-96222024v33e2023214.en

**Published:** 2024-02-19

**Authors:** Emanuelle Renck, Caroline Beatriz Zipper, Marcio Rodrigues Fabrino, Luisa Andrea Torres Salgado, Adriel Rowe, Ernani Tiaraju de Santa Helena

**Affiliations:** 1 Universidade Regional de Blumenau, Departamento de Medicina, Blumenau, SC, Brasil; 2 Universidade Regional de Blumenau, Programa de Pós-Graduação em Saúde Coletiva, Blumenau, SC, Brazil; 3 Prefeitura de Blumenau, Secretaria de Promoção da Saúde, Blumenau, SC, Brazil

**Keywords:** COVID-19 vaccines, Evaluation of Intervention Effectiveness, Severe Acute Respiratory Syndrome, COVID-19, Vacunas contra la Covid-19, Evaluación de Eficacia-Efectividad de Intervenciones, Síndrome Respiratorio Agudo Grave, Covid-19, Vacinas contra Covid-19, Avaliação de Eficácia-Efetividade de Intervenções, Síndrome Respiratória Aguda Grave, Covid-19

## Abstract

**Objective:**

to analyze the vaccine effectiveness in preventing deaths attributed to severe acute respiratory syndrome due to COVID-19 (SARS/COVID-19) in adults and the elderly, in Blumenau, state of Santa Catarina, Brazil, 2021.

this was a population-based study conducted among individuals aged 20 years and older hospitalized with SARS/COVID-19; each death due to SARS/COVID-19 was considered a “case”, and every survivor was considered a “control”; the association between vaccination status and the outcome of “death” was estimated using logistic regression, and vaccine effectiveness was estimated as (1-OR)*100.

The study included 1,756 cases of SARS/COVID-19 (59.2% male, mean age of 56 years, 50.4% with elementary education, 68.4% with comorbidities and 39.1% in intensive care), of whom 398 died (cases) and 1,358 survived (controls); vaccine effectiveness was 74% and 85% (20-59 years old) and 72% and 75% (≥ 60 years old), respectively, for those who were partially vaccinated and fully vaccinated.

**Conclusion:**

vaccines proved to be effective in reducing case fatality ratio due to SARS/COVID-19 in individuals ≥ 20 years old.

## RESUMEN

**Objetivo:**

analizar la efectividad de la vacuna para prevención de muertes por SRAG-COVID en adultos y ancianos de Blumenau, Santa Catarina, Brasil, 2021.

**Método:**

estudio de base poblacional con personas hospitalizadas por SRAG-COVID mayores de 20 años; las muertes por SRAG-COVID se consideraron casos y todos los supervivientes, controles; la asociación entre el estado de vacunación y la muerte se estimó mediante regresión logística; la efectividad de la vacuna se estimó por (1-OR)*100.

**Resultados:**

participaram do estudo 1.756 casos de SRAG-COVID (59,2% del sexo masculino, edad media de 56 años, 50,4% con estudios primarios, 68,4% con comorbilidades y 39,1% en cuidados intensivos), dos quais 398 foram a óbito (casos) e 1.358 sobreviveram (controles); la efectividad de la vacuna fue del 74% y el 85% (20 a 59 años) y del 72% y el 75% (60 y más años), entre los que tenían vacunación parcial y completa, respectivamente.

**Conclusión:**

las vacunas fueran efectivas para reducir la letalidad del SRAG-covid.

## RESUMO

**Objetivo:**

Analisar a efetividade vacinal na prevenção de óbitos atribuídos a síndrome respiratória aguda grave por covid-19 (SRAG-covid) em adultos e idosos, em Blumenau, Santa Catarina, Brasil, 2021.

**Métodos:**

Estudo populacional, entre maiores de 20 anos de idade internados por SRAG-covid; considerou-se “caso” cada óbito por SRAG-covid, e “controle”, todo sobrevivente; estimou-se a associação entre a condição vacinal e o desfecho “óbito” por regressão logística, e a efetividade vacinal, por (1-OR)*100.

**Resultados:**

Participaram do estudo 1.756 casos de SRAG-covid (59,2% do sexo masculino, idade média de 56 anos, 50,4% com ensino fundamental, 68,4% com comorbidades e 39,1% em cuidado intensivo), dos quais 398 foram a óbito (casos) e 1.358 sobreviveram (controles); a efetividade vacinal foi de 74% e 85% (20-59 anos) e de 72% e 75% (≥ 60 anos), respectivamente, para quem possuía vacinação parcial e vacinação completa.

**Conclusão:**

Vacinas mostraram-se efetivas na redução da letalidade por SRAG-covid em indivíduos com idade ≥ 20 anos.

## INTRODUCTION

Severe acute respiratory syndrome (SARS) is a diffuse and inflammatory form of lung injury, characterized by poor oxygenation, pulmonary infiltrates, and acute onset. Due to its characteristics and the life-threatening risk this disorder poses, much has been discussed about the occurrence of SARS attributed to the novel circulating virus, SARS-CoV^-^2.^1^ Cases of SARS-related hospitalization due to COVID-19 (SARS/COVID-19) reported in the Influenza Epidemiological Surveillance Information System (*Sistema de Vigilância Epidemiológica da Gripe* - SIVEP-Gripe) in Brazil in 2020 were 623,310, representing approximately 61.6% of all cases of SARS. In the following year, these cases increased by 16.7%; and 73.6% of SARS hospitalizations were due to COVID-^1^9.^2^

Globally, the number of deaths from the disease was estimated at 6.32 million as of June 20^2^2.^3^During the same period, Brazil recorded a total of 669,390 confirmed deaths due to COVID-19. In the Southern macro-region of the country, there were 105,346 deaths, and in the Southern state of Santa Catarina, specifically, 21,940 deaths were report^e^d.^4^ The five national macro-regions presented different case fatality rat^e^s,^1^ a factor likely supported by the diversity in the socioeconomic, cultural, and health characteristics of their populatio^n^s.^5^

In early 2021, a significant portion of the world began mass vaccination campaigns using newly approved vaccines against COVID-^1^9.^6^.^7^ Vaccination commenced in Brazil in January 2021, with the administration of the AstraZeneca/Fiocruz and Sinovac/Butantan vaccines; the Pfizer/Wyeth vaccine was included in May of the same year, and the Janssen vaccine in June, totaling four available vaccine products against the disease in the count^r^y.^4^ Evaluations of vaccine effectiveness are crucial not only to understand its effect on reducing infection and disease, but also to guide relevant public polici^e^s.^8^ Norway achieved a vaccine effectiveness in preventing deaths estimated at 46.9% after the first dose and 93.4% after the second do^s^e.^9^ An analysis of clinical trials for vaccines, using the World Health Organization (WHO) Emergency Use Listing, estimated variable vaccine effectiveness, ranging from 90% to 99% after two doses, against the “death” outcome. These values were lower after a single dose: 70% to 90% against the same outco^me^.^10^ A meta-analysis of records from 51 studies estimated vaccine effectiveness in preventing deaths from COVID-19 at 58.4% for the partial vaccination status and 98.1% for the full vaccination stat^us^.^11^ In Brazil, the vaccination effectiveness for the outcome of “death” ranged from 35.3% for individuals aged 80 years and older, with a partial vaccination schedule, to 84.5% in the age group of 40 to 59 years with a full vaccination schedu^l^e.^7^ According to another Brazilian study, conducted with national hospitalization and vaccination data, vaccine effectiveness against death from COVID-19 with full vaccination schedule ranged from 57.7% to 89.9%, while with a single-dose schedule, effectiveness ranged from 35.3% to 61.^8%^.^12^

Data on vaccine effectiveness against COVID-19 in reducing the risk of death in people with specific health conditions are scarce, such as in the case of people hospitalized with SARS/COVID-19 in Southern Brazil.

The objective of this study was to estimate vaccine effectiveness in reducing the risk of death in adults and older adults with SARS/COVID-19 living in the municipality of Blumenau, state of Santa Catarina, Brazil, in 2021.

## METHODS

This was a population-based case-control study on SARS/COVID-19 cases with onset of symptoms occurring between January 1 and December 31, 2021, in residents of Blumenau, state of Santa Catarina.

The municipality of Blumenau, state of Santa Catarina, founded by German immigrants in the Médio Vale do Itajaí (26° 55’ 08” South Latitude and 49° 03’ 57” West Longitude), had an estimated population of 366,418 inhabitants and a gross domestic product (GDP) per capita of BRL 48,416.09 in 2021. Blumenau is an important industrial, technological and university hub in the sta^te^.^13^

### Participants and Data Sources

All people with SARS/COVID-19 and a clinical picture of influenza-like illness who presented with dyspnea/respiratory distress or persistent chest pressure or oxygen saturation level below 95% on room air or blue discoloration of the lips or face, confirmed by RT-PCR te^st^,^14^ aged 20 years or older, living in Blumenau, with completed investigation and available in the SIVEP-Gripe database, were considered eligible for the study. The anonymized SIVEP-Gripe database was made available to researchers in April 2022.

**Figure 1:**
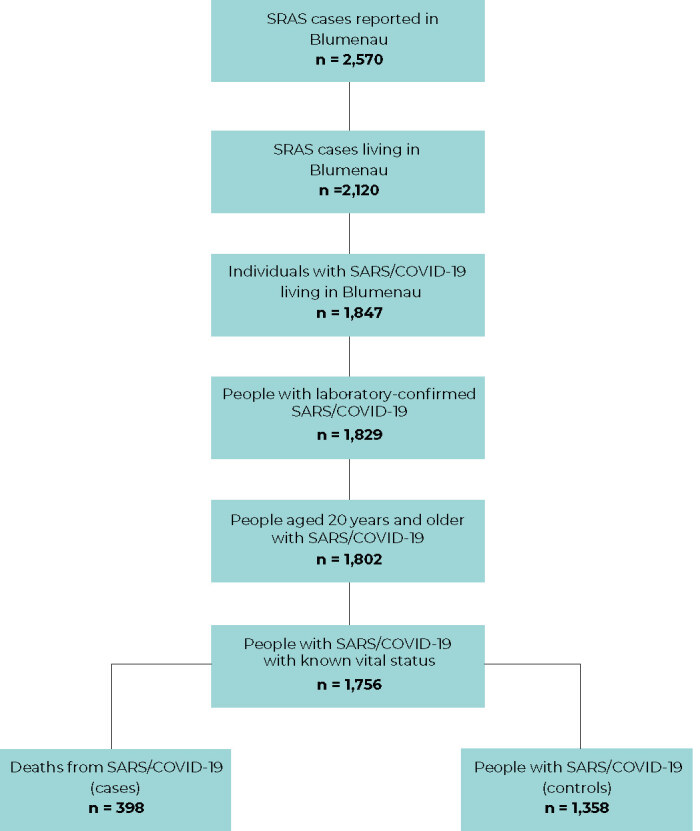
Flowchart of the selection of study participants, based on cases of severe acute respiratory syndrome due to COVID-19 (SARS/COVID-19), with onset of symptoms occurring between January 1 and December 31, in residents of the municipality of Blumenau, state of Santa Catarina, Brazil, 2021

“Cases” of SARS/COVID-19 resulting in death during the period established for the study were considered: n = 398. Based on the definition of death due to SARS/COVID-19 described here, additional information related to deaths in the System’s database was investigated by the Death Investigation Service of the Epidemiological Surveillance, in Blumenau, which checks the quality of the information recorded in Death Certificates (DCs) by reviewing outpatient and/or hospital medical records. All cases of SARS/COVID-19 that survived during the same study period were defined as “controls”: n = 1,360. Taking into consideration a first-dose vaccine coverage of 30% for 398 cases, and 40% for 1,358 controls, a study power of 97% was estimated for an odds ratio (OR) of 0.63 and a 95% confidence interval (95%CI). Furthermore, considering vaccine coverage with two doses or a single dose of 20% for 398 cases, and 30% for 1,358 controls, a study power of 98% was estimated for an OR = 0.58 and 95%CI (Figure 1).

Information on vaccination status (exposure variable) was reviewed at the Immunization Coordination of the Municipal Health Department of Blumenau.

### Exposure Variable

Regarding vaccination status, participants were classified as “unvaccinated”, “partially vaccinated” (received 1 dose) or “fully vaccinated” (received one dose of the single-shot Janssen vaccine, or received two doses of the others), regardless of vaccine type, brand or batch. In Blumenau, the first doses of vaccine were administered on January 20, 2021. Booster doses were not taken into account, as they only began from epidemiological week 37 of 2021, in the municipality.

### Confounding variables

The following adjustment variables, available in the database, were selected:

- sex (male; female);
- age group (in full years: 20 to 59; 60 to 79; 80 and older);
- self-reported race/skin color (White; non-White);
- schooling (illiterat^e;^ 1st ^to^ 5th grade of elementary schoo^l;^ 6th ^to^ 9th grade of elementary school; high school; higher education);
- place of residence (grouped neighborhoods);
- presence and number of comorbidities;
- type of comorbidity/risk factor (Down syndrome; diabetes *mellitus*; immunodeficiency; cardiovascular disease; chronic liver disease; chronic neurological disorders; chronic kidney disease; chronic hematologic disorders; asthma; other chronic lung diseases; obesity; and being a puerperal woman);
- influenza vaccination in the previous year (yes; no); and
- admission to the Intensive Care Unit (ICU) (yes; no).

The place of residence took into account the declared neighborhood, grouped by degree of urbanization according to the number of plots registered for agricultural use: (i) without any plots – Boa Vista, Bom Retiro, Centro, Itoupava Norte, Itoupava Seca, Jardim Blumenau, Ponta Aguda, Ribeirão Fresco, Victor Konder, Vila Formosa, Vila Nova and Vorstadt –; (ii) with up to 10 plots – Água Verde, Badenfurt, Da Glória, Do Salto, Escola Agrícola, Fortaleza, Garcia, Nova Esperança, Salto do Norte, Salto Weissbach, Tribess, Valparaíso, Velha, Velha Central and Velha Grande –; and (iii) with 10 to 20 plots – Progresso, Fidélis, Fortaleza Alta, Itoupava Central, Itoupavazinha, Passo Manso, Testo Salto and Vila Itoupa^va^.^15^

### Statistical methods

The variables were examined according to the type of distribution: continuous variables were presented by measures of central tendency and dispersion; and categorical variables, by absolute and relative frequencies. As these are secondary data, the presence of incompleteness was checked, and variables with more than 10% incompleteness were excluded.

The comparison of means was performed using Student’s t-test, and the comparison of proportions, using Pearson’s chi-square test. The association between vaccination status (partial; complete) and the occurrence of death, both overall and by age group, was estimated by means of odds ratio (OR) and 95%CI obtained using unconditional logistic regression, crude and adjusted for sex, age, schooling, place of residence, number of comorbidities, and previous influenza vaccination. All study variables with a p-value < 0.20 in the univariate analysis were included in the adjusted models.

Vaccine effectiveness was estimated using the following formula: 1 - OR of vaccination between cases and controls *1^00^.^16^ Model adjustment was estimated using the Hosmer-Lemeshow test. Statistical analyses were performed using the Stata 11.2. A p-value < 0.05 was considered statistically significant.

### Ethical aspects

This study is part of the research project entitled “COVID-19 vaccine effectiveness in Blumenau, state of Santa Catarina: a case-control study”, approved by the Human Research Ethics Committee (HREC), of the Fundação Universidade Regional de Blumenau (FURB), on August 7, 2021: Certificate of Submission for Ethical Appraisal (CAAE) No. 46513121.5.0000.5370; Opinion No. 4,891,763. Due to the use of secondary data, the project was exempt from requiring the participants to sign the Free and Informed Consent Form (FICF).

## RESULTS

There were 2,570 cases of SARS in residents of the municipality of Blumenau in 2021, and among them, 1,829 were confirmed as COVID-19 through RT-PCR test or antigen test. Of these, 1,756 people aged 20 years or older took part in the study, of whom 398 died from SARS/COVID-19 (cases) and 1,358 survived (controls). Figure 1 shows the participant selection flowchart.

Regarding data completeness for study variables, sex, age group, comorbidities, and COVID-19 vaccination status had 100% completeness. The percentage of incompleteness for the other variables was 5.2% for schooling, 4.2% for previous influenza vaccination, 2.5% for number of comorbidities, 2% for race/skin color, 1.5% for ICU admission, 0.5% for place of residence, and 0.2% for deaths.

Table 1 shows some of the clinical and epidemiological characteristics of the cases and controls. There was no statistically significant difference in sex and race/skin color between cases and controls. The mean age was higher among cases (66.3 versus 53.1 years; p-value < 0.001). In addition, a higher proportion of people with lower level of education, three or more comorbidities, without previous influenza vaccination and those who required ICU admission were observed among cases. As for associated comorbidities/risk factors, chronic cardiovascular disease (47.7%), obesity (35.6%), diabetes *mellitus* (31.9%), other chronic lung diseases, chronic kidney disease (4,2%), asthma (4,2%), chronic neurological disorders (3.6%), immunodeficiency (3.0%), chronic liver disease (0.8%), chronic hematological disorders (0.5%), being a puerperal woman (0.3%) and Down syndrome (0.1%) (data not shown in the table). Vaccination coverage, both for the first dose and for the second dose or single dose, was higher in the control group.

**Table 1:** Sociodemographic and clinical characteristics of cases of severe acute respiratory syndrome due to COVID-19 (SARS/COVID-19), according to cases (deaths) and controls (survivors), Blumenau, state of Santa Catarina, Brazil, 2021

Variables	Total n (%)	Cases n (%)	Controls n (%)	p-value^a^
**Sex (n = 1,756)**				
Female	716 (40.8)	179 (45.0)	537 (39.5)	
Male	1,040 (59.2)	219 (55.0)	821 (60.5)	0.050
**Age group in years (n = 1,756)**				
20-59	1,034 (58.9)	126 (31.7)	908 (66.9)	
60-79	572 (32.6)	189 (47.5)	383 (28.2)	
≥ 80	150 (8.5)	83 (20.8)	67 (4.9)	< 0.001
**Race/skin color (n = 1** **,723)**				
White	1,624 (94.3)	373 (94.4)	1,251 (94.2)	
Non-White	99 (5.7)	22 (5.6)	77 (5.8)	0.864
**Schooling (n = 1,667)**				
Higher education	327 (19.6)	35 (9.2)	292 (22.7)	
High school	500 (30.0)	84 (22.1)	416 (32.3)	
Illiterate/elementary education	840 (50.4)	261 (68.7)	579 (45.0)	< 0.001
**Grouped neighborhoods (n = 1,748)**				
Without any plots	378 (21.6)	83 (21.0)	295 (21.8)	
Up to 10 agricultural plots	810 (46.4)	165 (41.7)	645 (47.7)	
More than 10 agricultural plots	560 (32.0)	148 (37.3)	412 (30.5)	0.029
**Presen** **ce of comorbidities (n = 1,756)**				
No	555 (31.6)	25 (6.3)	530 (39.0)	
Yes	1,201 (68.4)	373 (93.7)	828 (61.0)	< 0.001
**Number of comorbidities (n = 1,713)**				
None	555 (32.4)	25 (6.4)	530 (40.0)	
One	468 (27.3)	90 (23.1)	378 (28.6)	
Two	413 (24.1)	139 (35.7)	274 (20.7)	
Three or more	277 (16.2)	135 (34.7)	142 (10.7)	< 0.001
**Influenza vaccine in the last campaign (n = 1,685)**				
No	993 (58.9)	182 (47.0)	811 (62.5)	
Yes	692 (41.1)	205 (53.0)	487 (37.5)	< 0.001
**ICU admission^b^ (n = 1,730)**				
No	1,054 (60.9)	107 (28.2))	947 (70.1)	
Yes	676 (39.1)	272 (71.8)	404 (29.9)	< 0.001
**Number of doses of COVID-19 vaccine**				
One	38.5%	30.2%	40.9%	< 0.001
Two or a single dose	27.3%	18.6%	29.8%	< 0.001

a) Pearson’s chi-square test; b) ICU: Intensive care unit.

Table 2 shows the results of the crude and adjusted analyses of the association between vaccination status and death, by dose and age group, in people with SARS. A higher chance of protection against death was found among vaccinated people, both among those who received one dose and those who received two doses.

**Table 2:** Crude and adjusted odds ratio (OR) of the association between vaccination status and deaths, by dose and age group, in people with severe acute respiratory syndrome due to COVID-19 (SARS/COVID-19), Blumenau, state of Santa Catarina, Brazil, 2021

Vaccination schedule (number of doses)	Total (n = 1,756)			Age group (in years)				
				**20 to 59 (n = 1,034)**			**60 and older (n = 722)**	
	**OR crude (95%CI )**	**OR adjusted** ^a^ **(95%CI)**		**OR crude (95%CI)**	**OR adjusted** ^a^ **(95%CI)**		**OR crude (95%CI)**	**OR ad justed** ^a^ **(95%CI)**
**One**	0.62 (0.48;0.79)	0.27 (0.20;0.38)		0.26 (0.15;0.47)	0.23 (0.12;0.43)		0.45 (0.33;0.61)	0.25 (0.17;0.37)
**Two or a single dose**	0.53 (0.40;0.71)	0.23 (0.16;0.33)		0.09 (0.03;0.28)	0.11 (0.03;0.35)		0.40 (0.29;0.56)	0.22 (0.15;0.34)

a) Odds ratio adjusted for sex, age group, schooling, neighborhood urbanization, risk factor, and influenza vaccine in the last campaign.

**Figure 2:**
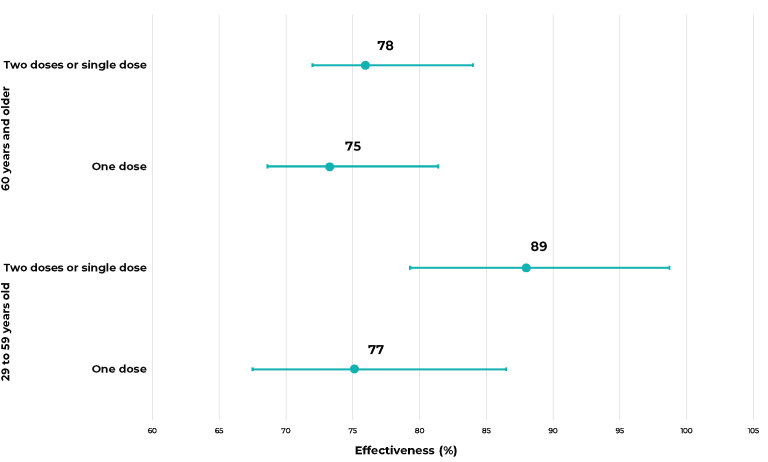
Vaccine effectiveness in reducing deaths in people with severe acute respiratory syndrome due to COVID-19 (SARS/COVID-19), by dose and age group, Blumenau, state of Santa Catarina, Brazil, 2021

Figure 2 presents the vaccine effectiveness in reducing deaths among people with SARS/COVID-19.

## DISCUSSION

Vaccination, adjusted for confounding variables, proved to be effective in reducing deaths from SARS/COVID-19. Even in populations aged 60 years or older and with incomplete vaccination status, vaccine effectiveness was above 70%.

When characterizing the study population in the clinical and epidemiological terms, it could be seen that the majority of people with SARS/COVID-19 were male, a finding similar to that of other natio^nal17^-19 and international studi^es^.^20^ The age group of 20 to 59 years was the most affected by SARS/COVID-19, consistent with findings from a national stu^dy^.^21^ However, SARS/COVID-19 hospitalizations were related to the oldest age groups, especially those aged 60 years and old^er.18^,^19^

In this sample from Blumenau, SARS/COVID-19 cases had a higher frequency of comorbidities when compared to controls. This finding is consistent with those of other studi^es.21^-23 The most prevalent comorbidities were cardiovascular disease, obesity, diabetes *mellitus,* and other chronic lung diseases unrelated to the current COVID-19 disease. The frequency of these comorbidities in cases of SARS-COVID-19 decreased significantly when compared to cases with mild COVID-^19^.^24^ It is also noteworthy that just under half of the individuals hospitalized due to SARS/COVID-19 required ICU admissions, a figure close to the national findin^gs^.^23^

When examining the profile of cases that progressed to death due to SARS/COVID-19, some peculiarities were observed, including the oldest age group (between 60 and 79 years), lower level of schooling (illiterate or with an education level up to elementary school) and the presence of comorbidities. The association between the risk of negative outcomes of COVID-19 and increasing age was identified at the beginning of the pandem^ic,24^,^25^ although it has not been extensively explored in the context of SARS/COVID-19. Lower levels of schooling were associated with deaths, both in Santa Catari^na^,^17^ and in Brazil as a who^le.18^-23 This can be explained, at least in part, by expressing educational and cultural characteristics associated with knowledge about the disease and its complications, as well as by representing an approximation of worse economic conditions.

In this study, vaccine effectiveness against SARS/COVID-19 showed a greater protective effect in the youngest age group of the sample, from 20 to 59 years old, and among those with the complete vaccination schedule, i.e., two doses or a single dose. In the state of Rio Grande do Sul, the State Center for Health Surveillance found 58% effectiveness of partial COVID-19 vaccination in the occurrence of SARS/COVID-19 among the elderly; and over 90% in the population with a complete vaccination schedule, among those aged 20 to 59 yea^rs^.^26^ Consistent with the findings of this study, it could be seen that the estimates of vaccine effectiveness in preventing deaths are higher in the younger age gro^u^p.^7^ Such differences in vaccine effectiveness are likely attributed to the different vaccination schedules (the complete vaccination schedule provides greater stimulus to the immune response) and the different age groups [in older adults, the immunosenescence process (changes in the immune system caused by aging), combined with the presence of comorbidities, hinders the immune respons^e].9^,^27^

As the study used secondary data, it was subject to information bias, given that the data may contain diagnostic errors, failures, and incompleteness in the records. In addition, the effectiveness by type of vaccine administered was not analyzed, since this information was not available in the database used at the time of the study. Nevertheless, this study depicted real-world vaccine effectiveness and its use conditions.

It can be concluded that vaccination against COVID-19 proved to be effective in reducing the risk of death among people aged 20 years and older with SARS/COVID-19 in Blumenau in 2021. It is recommended to expand vaccine coverage and booster doses as strategies to prevent deaths due to SARS/COVID-19, especially among the population aged 60 years and older, with low level of schooling and the presence of comorbidities.
